# CRISPR-Cas13b mediated gene knockdowns in *Leishmania infantum*

**DOI:** 10.1016/j.ijpddr.2025.100629

**Published:** 2025-12-05

**Authors:** Marine Queffeulou, Raouia Fakhfakh, Fereshteh Fani, Alex Dos Santos, Gabriel Reis Ferreira, Sophia Bigot, Chantal Godin, Philippe Leprohon, Barbara Papadopoulou, Marc Ouellette

**Affiliations:** Centre de Recherche en Infectiologie du Centre de Recherche du CHU Québec and Département de Microbiologie, Infectiologie et Immunologie, Faculté de Médecine, Université Laval, Québec, Québec, Canada

**Keywords:** CRISPR-Cas13, *Leishmania*, Loss-of-function, Drug resistance, Miltefosine transporter, Aquaglyceroporin 1

## Abstract

Chemotherapy is an effective means to control infections caused by the protozoan parasite *Leishmania*. However, available treatments are limited, expensive, and associated with considerable toxicity. Genomic strategies have contributed to a better understanding of *Leishmania*'s response to drugs and in the characterization of drug targets. Nonetheless, there is no knockdown system operational for *Leishmania*. In this study, we show that the CRISPR-Cas13 system can be an effective strategy to knockdown expression levels of both exogenous and endogenous transcripts. We succeeded in effectively knocking down the expression of the firefly luciferase gene integrated in the genome of *L. infantum*. This Cas13-mediated decrease in mRNA was paralleled with a significant reduction in both the luciferase protein level and its activity. Furthermore, we tested the effectiveness of the Cas13 system to target the endogenous miltefosine transporter (*MT*) and the aquaglyceroporin 1 (*AQP1*) genes. Knockdown was effective and parasites with less *MT* or *AQP1* mRNA levels exhibited reduced susceptibility to miltefosine or antimonials, respectively. While further optimization is warranted, this knockdown system has the potential to facilitate numerous studies related to various aspects of *Leishmania* biology.

## Highlights

CRISPR-Cas13 system:

• Demonstrates the successful application for gene knockdowns in *Leishmania*
• Enables transient and specific silencing without altering genomic DNA
• Opens opportunities for high-throughput studies, essential gene analysis

## Graphical abstract

**Figure:**
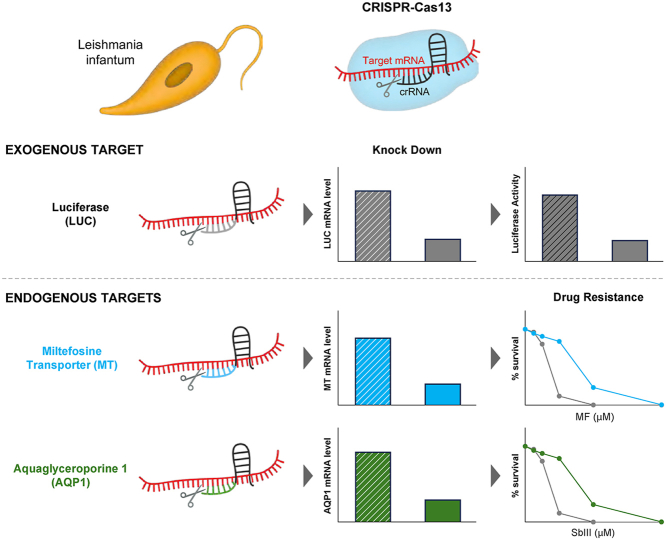

## 1 Introduction

Leishmaniasis is a group of infectious diseases caused by parasites belonging to the genus *Leishmania* that is endemic in 98 countries. With an estimated prevalence of 700,000 to 1 million new cases each year (World Health Organization, 2023), leishmaniasis is a major public health problem in several continents. However, the absence of vaccines for humans and the limited effectiveness of sand fly vector control programs, emphasize the current necessity of chemotherapy to control the various forms of the disease (Dinc, 2022). Moreover, available treatments are limited, expensive, and associated with considerable toxicity or drug resistance. The commonly used compounds are limited to pentavalent antimonials, amphotericin B and its formulations, miltefosine (MF), paromomycin sulfate, and more rarely, pentamidine isethionate (Brindha et al., 2021). New drugs are therefore urgently needed.

The sequencing of the first *Leishmania* genome (Ivens et al., 2005) led to a wind of optimism in our search for new drug targets but its analysis revealed a significant number of annotated proteins of unknown function. Molecular strategies based on transcriptomic (Do Monte-Neto et al., 2011; Cantacessi et al., 2015; Rastrojo et al., 2018), proteomics (Chawla et al., 2011; Walker et al., 2012; Brotherton et al., 2014; Rabaan et al., 2022), metabolomics (t’Kindt et al., 2010; Vincent et al., 2014; Armitage et al., 2018), and large-scale sequencing (Downing et al., 2011; Leprohon et al., 2015) have contributed to a better understanding of *Leishmania* response to drugs, and in the characterization of new drug targets (Rivas and Gil, 2017; Van Den Kerkhof et al., 2020). Whole genome screens based on gain or loss of functions (Gazanion et al., 2016; Fernandez-Prada et al., 2018; Bhattacharya et al., 2019; Potvin et al., 2023; Queffeulou et al., 2024; Albuquerque-Wendt et al., 2025; Herrmann May et al., 2025) have proven a useful complement for the identification of resistance mechanisms and novel therapeutic targets. These screens mostly lead to point mutations or change in copy number (gene amplification or deletion) that could be detected most easily by genomic DNA sequencing.

RNA-based screens such as RNAi, siRNAs, miRNAs have permitted formidable advances in many spheres of life sciences (Fire et al., 1998; Agrawal et al., 2003). For example, RNAi is the method of choice for knockdown approaches in *Trypanosoma brucei* (Ngô et al., 1998; Alsford et al., 2011; Collett et al., 2019), a parasite distantly related to *Leishmania*. Indeed, RNAi screens have been most useful in the discovery in drugs’ mode of action in *T. brucei* (Ngô et al., 1998). RNAi, however, is not functional in the parasite *Leishmania*, although intriguingly it is active in the South American *Viannia* subgenus (Lye et al., 2010; Atayde et al., 2013; De Paiva et al., 2015). Clustered regularly interspaced short palindromic repeats (CRISPR)-associated proteins (Cas) systems are naturally present in bacteria and archaea (Barrangou et al., 2007; Marraffini and Sontheimer, 2010). The CRISPR-Cas systems have been categorized into two classes, six types, and 33 subtypes, differing in many aspects, including the target substrates (Hryhorowicz et al., 2023). The class 2, type II CRISPR-Cas9 system has rapidly emerged as a tool for functional research in the parasite *Leishmania* (Sollelis et al., 2015; Zhang and Matlashewski, 2015; Engstler and Beneke, 2023; Asencio et al., 2024; Queffeulou et al., 2024). A new class 2 type VI RNA-guided RNA-targeting CRISPR–Cas effector, Cas13a (formerly C2c2), from *Leptotrichia wadei* has been engineered in eukaryotic and prokaryotic cells for RNA knockdowns, base editing, and binding/tracking/detection without modifying their genomes (Abudayyeh et al., 2016, 2017; Cox et al., 2017; Gootenberg et al., 2017). Cas13 proteins have two distinct ribonuclease activities: one that processes the pre-crRNA, and two Higher Eukaryotic and Prokaryotic Nucleotide (HEPN)-binding domains that activate the degradation of cis- and trans-targeted single-stranded RNA. Five subtypes of Cas13 nucleases have been described and interestingly they do not require a specific PAM sequence but a longer crRNA (Abudayyeh et al., 2016; East-Seletsky et al., 2016; Huynh et al., 2020). Over 42 Cas13 orthologs (subtypes a, b, and c) were assessed for their RNA knockdown activity and stability with a gRNA targeting the luciferase gene in human embryonic kidney (HEK) 293FT cells (Cox et al., 2017). The Cas13b (formerly c2c6) ortholog from the species *Prevotella sp.* P5-125 was associated with the highest level of luciferase knockdown (Cox et al., 2017). Cas13b also demonstrated the highest specificity and sensitivity to mismatches against its target (Huynh et al., 2020).

In this study, we have tested the CRISPR-Cas13b system as a tool for knocking down specific RNA sequences in *Leishmania infantum*.

## 2 Materials and methods

### 2.1 Parasite culture and strains

Wild type (WT) *Leishmania* infantum (MHOM/MA/67/ITMAP-263) parasites were cultured as promastigotes at 25 °C in SDM-79 medium supplemented with 10 % (vol/vol) heat-inactivated fetal bovine serum (FBS), 10 μM biopterin and 5 μg/mL hemin at pH 7. Selection drugs were added to the medium at the following concentrations: 600 μg/mL hygromycin B (Wisent, Multicell), 80 μg/mL puromycin, 40 μg/mL G418 disulfate (Sigma-Aldrich), 25 or 40 μM Miltefosine (MF, Cayman Chemical), and 375 or 750 μM of potassium antimonial tartrate (SbIII). Miltefosine and antimonial resistance was determined by measuring the OD600 nm after 72 h, as described previously (Ouellette et al., 1990). The strain LUC-3′UTR4000 cl2 (Azizi et al., 2017) was obtained by integrating the firefly luciferase (*LUC*) gene into the gene encoding a mitochondrial aminomethyltransferase (LINF_360047000) of *L. infantum*.

### 2.2 Construction of Cas13 and gRNA vectors

**Fig. 1:**
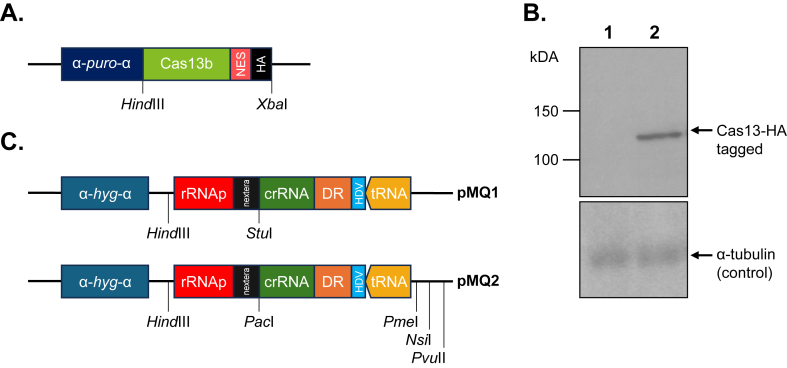
**Vectors for CRISPR-Cas13b mediated gene knockdowns in *Leishmania infantum*. A. Schematic map of the Cas13b expression vector.** A PCR fragment containing the Cas13b gene fused to a nuclear export signal (NES) and 3xHA tags (39) was cloned in pSP72 α-PURO-α (47); α corresponds to the intergenic region of the *Leishmania* α-tubulin gene cluster (48) and is sufficient for proper gene expression in *Leishmania*. **B. Expression of Cas13b in *L. infantum***. Western blot of *L. infantum* protein extracts reacted with an anti-HA antibody. 1, *L. infantum* WT; 2, *L. infantum* transfected with the Cas13b vector. Marker bands are annotated. The blot was stripped and reacted with an α-tubulin antibody to monitor the amounts of proteins layered in each lane. **C. Schematic map of the gRNA vectors.** The expression of the gRNAs in pMQ1 and pMQ2 is driven by a cassette including a ribosomal promoter (rRNAp) and transcription terminators including a hepatitis delta virus (HDV) ribozyme sequence and a tRNA in antisense orientation. An Illumina (Nextera) adapter came from backbone vectors used in the generation of pMQ1. These cassettes were cloned in the pSPα-HYG-α (51) vector. More details about the generation of the final constructs can be found in Fig. S1.

We amplified the Cas13b gene by PCR (using primers C and D shown in Table S1) from a plasmid (Addgene, #103862) containing the gene derived from *Prevotella sp.* P5-125 tagged with 3xHA (Cox et al., 2017). This fragment was cloned into the *Hin*dIII and *Xba*I restriction sites of the expression vector pSP72-ɑ-PURO-ɑ harboring the selectable marker puromycin N-acetyltransferase gene (*PURO*)(Coelho et al., 2012b) and the alpha-tubulin intergenic sequences for proper gene expression in *Leishmania infantum* (Laban et al., 1990) (see Fig. 1A). We generated a DNA synthetic cassette containing i) a 26 bp crRNA luciferase sequence; ii) a 88 bp sequence derived from the genome of *Bergeyella zoohelcum* ATCC 43767 with Direct Repeat (DR) sequences that form a hairpin loop analogous to the Cas9 tracrRNA system and shown to be effective in Cas13b knockdown studies (Smargon et al., 2017); and iii) two transcriptional terminators: a 68-bp hepatitis delta virus (HDV) ribozyme sequence (Zhang and Matlashewski, 2015; Gardiner and Kazan, 2018) and Lys-tRNA in antisense orientation (Queffeulou et al., 2024) that were flanked by *Stu*I-*Cla*I restriction sites (Fig. S1). This synthetic cassette was cloned into the *Stu*I-*Cla*I sites of the DD vector (Queffeulou et al., 2024). The insert of this vector was amplified by PCR and cloned into the pSPɑHYGɑ (Fadili et al., 2002) *Pvu*II site (Fig. S1) leading to pMQ1 (Fig. 1B). This plasmid was not optimal however for the cloning of multiple gRNA cassettes and we thus generated pMQ2 (Fig. 1B). pMQ2 was made by first PCR amplifying the pMQ1 *Leishmania* ribosomal RNA (rRNA) promoter (Boucher et al., 2004) and its Nextera adapter while adding multiple unique restriction sites, including *Pac*I and *Pme*I. This PCR fragment was cloned into the pSPɑHYGɑ vector (Fig. S1). Other gRNAs were amplified by PCR following a similar protocol as *LUC* (primers 1 to 10 and *Pme*I-tRNA-RV; Table S1) but designed with 28 bp crRNA (except for AQP1 which was 26 bp as the *LUC* crRNA) integrated into the pSP-rRNAp vector by *Pac*I and *Pme*I insertions (Fig. S1).

### 2.3 RNA structure predictions

The secondary structures of the mRNA of the targeted genes were predicted using the RNAfold web server software from the ViennaRNA Web Services (Gruber et al., 2008). The choice of crRNAs mainly targeted sequences whose putative conformation was predicted to be easily accessible by the Cas13b protein. A crRNA complementary to the 5′end of the *LUC* sequence (from position 15 to 41), as well as to the middle of the gene (from position 1077 to 1103) were designed. Nine crRNAs were designed against the LINF_130020800 phospholipid-transporting ATPase 1-like protein *MT* gene, whose functional inactivation is known to lead to MF resistance (Pérez-Victoria et al., 2003a; Coelho et al., 2012a; Zhang and Matlashewski, 2015).

### 2.4 Transfections and drug selection

Transfections of *L. infantum* promastigotes were done by nucleofection using the Amaxa™ 4D-Nucleofector™ (Lonza) with the 3 Primary Cell 4D-Nucleofector™ X Kit and the Unstimulated Human T Cells program, as recommended by the manufacturer. Specifically, 8–15 μg of DNA were used with 5 × 10^7^ cells per transfection. After nucleofection, the parasites were allowed to recover for 24 h, pre-selected with half of the selection marker concentration, and 48 h later, 1 ml culture was passaged in fresh medium with appropriate drug selection. Mock controls corresponded to transfections with no DNA or control vectors. When indicated, cells were selected with MF or SbIII. Once the transfected cells reached an O.D of 0.5, 5 × 10^7^ cells were incubated into 10 mL of fresh SDM medium, 5 μL of 10 mM biopterin and 40 μM Miltefosine (MF, Cayman Chemical) or 750 μM SbIII.

### 2.5 Luciferase *in vitro* assay and detection

Luciferase assay was performed with the Luciferase Assay System (Promega Corporation) according to the manufacturer's instructions. Briefly, 1 × 10^7^ *L. infantum* promastigotes of *LUC*-3′UTR4000 cl2 derived lines were harvested at log-phase, washed, and resuspended in lysis buffer. The resulting cell lysates were mixed with substrate prior to luminescence measurement. Luminescence was measured in a 96-well plate luminometer system (MLX Microtiter; Dynex Technologies) recording relative light units (RLUs) with a total read time of 10 s. The activity was measured in 3 biological replicates. For detecting the LUC protein, 1 × 10^8^ promastigotes of *L. infantum LUC*-3′UTR4000 cl2 derived lines at late log phase were collected, centrifuged 5 min at 1150 *g*, resuspended with 100 μL of 5x Laemmli buffer and heated 5 min at 100 °C. The equivalent of 15 × 10^6^ cells were loaded on 12 % SDS-polyacrylamide gels and transferred to Hybond nylon membranes (Amersham) and reacted with the goat anti-luciferase antibody (1:1000; Fisher) and the anti-goat secondary antibody (1:10000; Santa Cruz biotechnology). The LUC protein was revealed by chemiluminescence (Western Lightning® Plus-ECL; PerkinElmer). To monitor the amount of protein layered in the gel, the membrane was stripped and reacted with the mouse anti-ɑ-tubulin (1:10000; Sigma), and the anti-mouse secondary antibody (1:10000; Cell Signaling Technology).

### 2.6 RNA analysis

Total RNA was extracted from 10 mL of *L. infantum* promastigotes in mid-log phase after lysis with 1 mL of Trizol (Invitrogen), following manufacturer's instruction. For Northern blot analysis, 20 μg of total RNAs were electrophoresed and transferred to nitrocellulose membranes (Hybond XL; Amersham). Radioactive probes complementary to open reading frames of the luciferase and glyceraldehyde-3-phosphate dehydrogenase (*GAPDH*, LINF_360030600) genes (Table S1) were amplified by PCR and labeled using [α-^32^P] dCTP, random oligonucleotides and the Klenow enzyme (New England Biolabs).

For qRT-PCR, RNA from promastigote mid-log phase was extracted in triplicates using the RNeasy plus mini kit (Qiagen), following the manufacturer instructions. The cDNAs were synthesized using Oligo (dT)_12-18_ primers (Invitrogen) and the SuperScriptII™ RNase H^−^ Reverse Transcriptase (Invitrogen). The qRT-PCR amplification was performed on a Rotor Gene RG-3000 and RG-6000 (Corbett research), using iQ™ SYBR® Green Supermix (Bio-Rad) and primers listed in Table S1. Ten μL reactions were run with 4 min of denaturation at 95 °C followed by 40 cycles of 20 s at 94 °C*,* annealing at 58 °C for 20 s and a final step extension at 72 °C for 20 s. The RNA expression levels were determined for each biological replicate from at least three technical replicates and were normalized to the constitutively expressed *GAPDH* mRNA. Standard curves used for RNA concentration were calibrated under the manufacturer's recommendations and according to the following parameters: coefficient of correlation R^2^ around >0.99, slope (M) around −3.3 and efficiency between 90 and 110 %. A maximum of 0.2 Ct was accepted between technical triplicates.

### 2.7 Statistical analysis

Data were statistically analyzed by the GraphPad Prism 5.1 software using two-tailed unpaired *t*-test. The data represent mean ± standard deviation (SD) of at least three replicates from at least two independent experiments. A *P* value of <0.01 was considered significant.

## 3 Results

### 3.1 Optimizing the Cas13b system in *L. infantum* using the exogenous *LUC* firely luciferase as a target gene

We first constructed a vector compatible with the constitutive expression in *L. infantum* of the Cas13b protein derived from *Prevotella sp.* P5-125 in (Fig. 1A). This vector was stably expressed in *L. infantum*, and Cas13b HA-tagged expression was monitored by western blot using an anti-HA antibody. A protein of the expected molecular weight was indeed detected in the Cas13b transfectants and not in control cells (Fig. 1B). We have also shown that *L. infantum* overexpressing constitutively Cas13b had no growth defect (Fig. S2). To optimize the Cas13b system, we used the exogenous *LUC* firely luciferase as a target gene (Cox et al., 2017; Slaymaker et al., 2019; Tng et al., 2020). To set up the Cas13b system in *L. infantum* we therefore started with the strain LUC-3′UTR4000 cl2 where *LUC* was integrated as a single copy in the genome and was shown to be functional (Azizi et al., 2017). We generated a vector, pMQ1 (Fig. 1C; see Fig. S1 for details) that encodes a 26 bp *LUC* crRNA (primers A and ClaI-tRNARV; Table S1) fused to a sequence recognized by Cas13b under the control of the rRNA *Leishmania* promoter (Boucher et al., 2004). This crRNA is a reverse complement sequence of a region of the 5′-end (nucleotides 15 to 41) of the *LUC* gene where we attempted to exclude regions putatively involved in complex secondary structures such as stem-loops or pseudoknots (Fig. S3). The plasmid pMQ1 was transfected into the strain LUC-3′UTR4000 cl2 that was previously transfected with the Cas13b-PURO vector (Fig. 1A), or in cells with no Cas13b plasmid. Cells growing in the presence of hygromycin (the marker of the gRNA vector) were passaged once, and their DNA, RNA, proteins and cell extracts were isolated and analyzed.

**Fig. 2:**
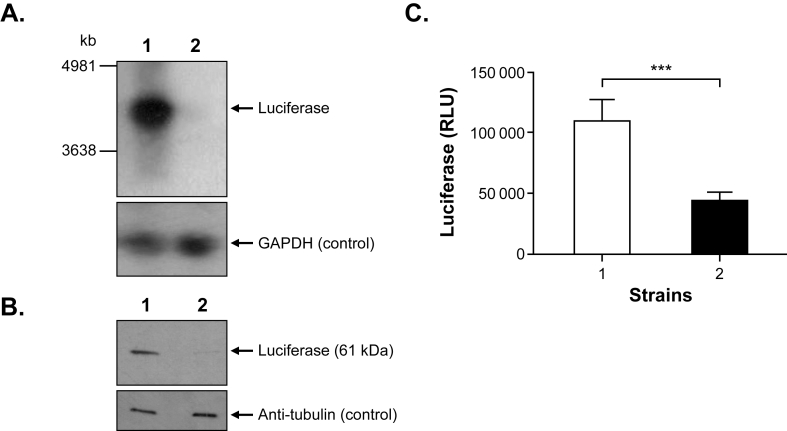
**CRISPR-Cas13b mediated gene expression knockdown in *Leishmania infantum*.** The recombinant *Leishmania infantum*-3′UTR4000cl2 strain (46) in which the *LUC* gene was integrated into the genome (*LINF_360047000;* aminomethyltransferase - mitochondrial) expressing the pMQ1 containing the gRNA LUC-A expression vector was transfected without (1) or with (2) the Cas13b expression vector (see Fig. 1A). **A. RNA expression**. The effect of CRISPR-Cas13b system on *LUC* mRNA expression was monitored by Northern blot. Ten μg of total RNA were extracted from *L. infantum* lines at mid-log phase and probed with PCR-generated probes (see Table S1 for primers) targeting the *LUC* and *GAPDH* genes. Molecular weight standards are indicated. **B. Protein expression.** To assess protein expression, 1 × 10^8^ cells were extracted and lysed from *L. infantum* transfectants at log phase. 15 × 10^6^ cells were loaded per well on SDS-PAGE gels and blotted on Hybond**™** nylon membranes and incubated with antibodies against luciferase (1/1000), stripped and reacted with α-tubulin (1/10000) antibody as a control. Protein expression was visualized by the ECL detection system. **C. Luciferase activity**. Luminescence analysis was done on 1 × 10^7^ cells of *L. infantum* transfectants using a 96-well plate luminometer system. Data represent the mean relative light units (RLUs) from 3 independent experiments with technical triplicates. Error bars indicate standard deviation. Significance was denoted as ∗∗∗p ≤ 0.0001. Luciferase activity was negligible (0.25 ± 0.02 RLU) in WT cells transfected with the Cas-13 vector. For A, B and C: lane 1, *L. infantum* LUC-3′UTR4000Cl2 transfected with PMQ1; lane 2, *L. infantum* LUC-3′UTR4000Cl2 transfected with PMQ1 and the Cas13b vector (see Fig. 1A).

Given the RNA endonuclease activity of Cas13b, it was expected that cells expressing Cas13b with the proper gRNA would lead to a specific decrease of the *LUC* mRNA. This was tested by Northern blot analysis. A band of the expected size (*LUC* and 3′ untranslated region) was observed in strain LUC-3′UTR4000 cl2 transfected with pMQ1 but the level of *LUC* mRNA was drastically diminished in the LUC-3′UTR4000 cl2 strain transfected with both the Cas13b-PURO vector and pMQ1 (Fig. 2A). The amount of RNA layered in each lane was controlled by the hybridization, to a stripped blot, with a probe recognizing the *GAPDH* transcript (Fig. 2A). This decreased *LUC* mRNA expression was paralleled by a decreased in LUC protein expression. Indeed, proteins derived from the two lines were reacted with a luciferase antibody and the signal of the LUC protein was 3-fold lower in the LUC-3′UTR4000 cl2 strain expressing both the Cas13b-PURO and pMQ1 vectors compared to the control line, once normalized with an antibody against alpha-tubulin (Fig. 2B). This decreased LUC RNA and protein expression was paralleled with a decreased luciferase activity, as measured by a luminometer (Fig. 2C). To exclude nonsense or missense mutations at the genomic level, the *LUC* gene in the LUC-3′UTR4000 cl2 strain expressing both the Cas13b-PURO and pMQ1 vectors was amplified and its Sanger sequencing revealed no mutations. As these Cas13b results are unprecedented for *Leishmania*, we carried out an independent electroporation of pMQ1 in the Cas13b expressing *L. infantum* recombinant strain, and we found that the LUC activity was similarly decreased in this new independent transfectant (Fig. S4). We also tested an additional gRNA (primers B and ClaI-tRNARV; Table S1), targeting a different region of the *LUC* gene (nucleotides 1077 to 1103) and a decreased in luciferase activity was also observed, albeit the decrease in activity was less significant in comparison to the initial guide tested at the 5′end of the gene (Fig. S4).

### 3.2 Efficient knockdown of endogenous *Leishmania infantum* transcripts using the Cas13b system

**Fig. 3:**
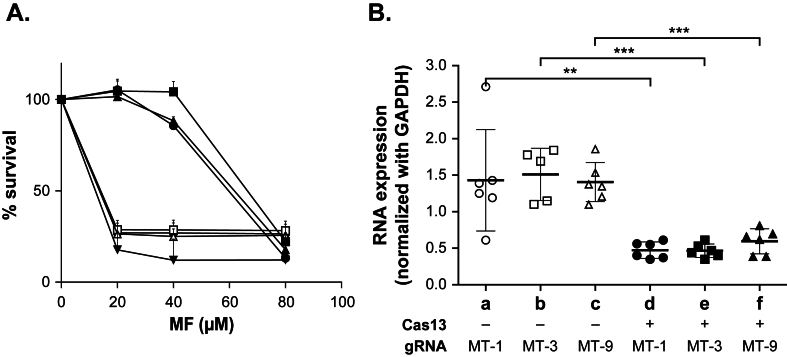
**CRISPR-Cas13b mediated knockdown of the *L. infantum* miltefosine transporter *MT* gene.** Cells expressing the Cas13b vector (black) or not (white) were transfected with pMQ plasmids encoding the gRNAs MT1 (◯, ●), MT-3 (☐, ■) or MT-9 (△, ▲), or LUC-A (▼) **A. Miltefosine susceptibility**. Cells expressing both Cas13b and MT gRNAs could be selected with 5X EC_50_ miltefosine. The EC_50_ values were calculated by monitoring their growth when incubated in presence of gradual MF concentrations, at 600 nm at the 72h end-point or when the baseline (cells without MF) reached an O.D of 0.5. Cells not expressing Cas13b or the ones transfected with the *LUC-*A gRNA could not be selected with 5X EC_50_ miltefosine and their EC50's were measured after transfections of the MQ MT-plasmids. The mean and standard deviation of a minimum of two independent experiments in technical triplicates are shown. **B. Monitoring *MT* mRNA expression**. Quantitative real-time RT-PCR was conducted to measure *MT* gene expression, normalized against the constitutively expressed *GAPDH* gene. The presence or not of Cas13b and the presence of distinct *MT* gRNAs are indicated below the graph. RNA extractions were performed from mid-log phase promastigotes in biological triplicates, with RNA concentration measurements assessed in technical triplicates represented here by the mean. Standard deviation is represented by error bars and significance was denoted as ∗∗p ≤ 0.001 and ∗∗∗p ≤ 0.0001.

Since the Cas13b system was found to be operational with the *LUC* gene (Fig. 2), we next selected to target the endogenous phospholipid-transporting ATPase 1-like protein (LINF_130020800) *MT* gene, coding for the miltefosine transporter. A *MT* knockdown is expected to lead to decreased MF susceptibility. We cloned 9 different crRNAs targeting the *MT* gene by PCR with *Pac*I-*Pme*I sites (Fig. S1) leading to gRNA encoding plasmids comparable to pMQ2 with only the crRNAs differing. These 9 crRNAs were reverse complement sequences spread along the *MT* coding sequence (Fig. S5). These pMQ2-like plasmids, along the gRNA pMQ1 against *LUC* as a control (A; Table S1), were transfected individually in strains expressing or not Cas13b. Parasites growing in the presence of hygromycin alone or hygromycin and puromycin were passaged and grown with 40 μM of MF. None of the strains transfected with pMQ2 plasmids containing crRNAs targeting *MT* grew in the absence of Cas13b (Fig. S6). Similarly, the strain transfected with pMQ1 (targeting *LUC*) and Cas13b also failed to grow in presence of MF (Fig. 3A and S6). Of the nine lines co-transfected with pMQ2 plasmids containing crRNAs targeting *MT* and the Cas13b-expressing plasmid, seven strains grew in the presence of 40 μM MF, while two strains with the MT-2 and MT-5 gRNAs (Fig. S6; Table S1) did not. We observed variations in the capacity of the 7 crRNA sequences to target *MT* permitting growth in the presence of 40 μM MF. The most effective was the one with the MT-1 gRNA that grew in eight days, followed by MT-8 and MT-9, then MT-3. One week later, lines expressing the MT-6, MT-7, and MT-4 gRNAs also grew. We selected strains with the MT-1, MT-3, and MT-9 gRNAs for more detailed analysis. These cells showed an increased EC_50_ to MF (Fig. 3A) and this resistance was due to a knockdown of the expression of the *MT* gene as measured by RT-qPCR (Fig. 3B). Since mutations in *MT* can also lead to MF resistance (Pérez-Victoria et al., 2003b; Laffitte et al., 2016; Coelho et al., 2012a), we amplified the *MT* gene from *L. infantum* WT or Cas13b-expressing strains with the MT-1/MT-3/MT-9 gRNAs and confirmed the absence of mutations in *MT*. Of note, MF selection from the onset is essential for observing our phenotype, as cells transfected with MT gRNAs and selected and passaged only with puromycin and hygromycin did not show an increased EC_50_ for MF. A similar result was observed when we performed an additional independent transfection with PMQ2 plasmid encoding MT-1 gRNA in *L. infantum* expressing Cas13b. Cells selected with hygromycin and puromycin alone (control) displayed an EC_50_ of 20 μM for miltefosine similarly to wild-type cells (Fig. S7A). In contrast, cells exposed to 60 μM miltefosine for three passages followed by three passages without miltefosine exhibited a two-fold increase in resistance to miltefosine (Fig. S7A). This decreased in susceptibility was correlated with a reduction in *MT* mRNA as determined by qRT-PCR (Fig. S7B).

**Fig. 4:**
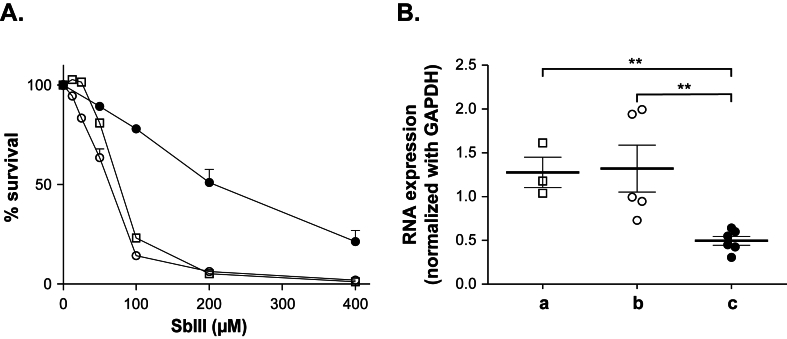
**CRISPR-Cas13b mediated knockdown of the *L. infantum* aquaglyceroporin gene *AQP1*.** Recombinant *L. infantum* cells expressing the Cas13b vector and transfected with pMQ2-derived plasmids encoding the gRNA against AQP1 grown without SbIII selection (◯, b) or with 10 X EC_50_ of SbIII for 3 passages followed by 3 passages without SbIII (●, c). Cells transfected with the Cas13b vector alone (☐, a) did not grow in the presence of SbIII. **A. Antimonial (SbIII) susceptibility**. The EC_50_ values were calculated by monitoring their growth when incubated in presence of gradual SbIII concentrations, at 600 nm at the 72h end-point. The mean and standard deviation of a minimum of three biological replicates are shown. **B. Monitoring *AQP1* mRNA expression**. Quantitative real-time RT-PCR was conducted to measure *AQP1* gene expression, normalized against constitutively expressed *GAPDH* gene. RNA extractions were performed from mid-log phase promastigotes in biological triplicates, RNA concentration measurements assessed in at least three independent experiments with technical triplicates and represented here by the mean. Standard deviation is represented by error bars and significance was denoted as ∗∗p ≤ 0.001.

Next, we tested whether this knockdown approach could be replicated to other genomic loci and we selected the aquaglyceroporin gene 1 *AQP1* whose downregulation is known to correlate with antimony resistance (Marquis et al., 2005). The gRNAs were amplified by PCR (primers 10 and *Pme*I-tRNA-RV; Table S1), digested and cloned into the *Pac*I and *Pme*I sites of pMQ2 (Fig. 1). Transfection of PMQ2 (with gAQP1-26 nt) and Cas13b had the same EC_50_ towards SbIII as control cells (Fig. 4A). When cells were grown with 750 μM SbIII for three passages, followed by three passages without SbIII, they were 3-fold more resistant to SbIII (Fig. 4A). This decreased in SbIII susceptibility was correlated with a significant reduction in *AQP1 m*RNA levels (Fig. 4B) as determined by qRT-PCR. Note that control cells without the PMQ2 plasmid did not grow when selected with SbIII.

## 4 Discussion

While several functional molecular tools are now available for *Leishmania*, we are still lacking a strategy for knocking down gene expression. Indeed, strategies such as RNAi have shown their usefulness when applied to different parasites (Geldhof et al., 2006; Rosso et al., 2009; Marie et al., 2015; Stortz et al., 2017; Marques et al., 2022) including the South American *Leishmania* subgenus *Viannia* (Lye et al., 2010; Atayde et al., 2013; De Paiva et al., 2015). Here, we demonstrated that the CRISPR-Cas13b system can serve as a tool for knocking down the expression of genes and validated the system with *LUC*, *MT* and *AQP1* mRNAs (Fig. 2, Fig. 3, Fig. 4). This is a first step demonstrating the feasibility of using Cas13b in *Leishmania* for knockdown purposes. Conventional CRISPR-Cas9 mediated knockouts in *Leishmania* can lead to allele deletions and thus inhibition of gene expression. This, however, can be challenging for essential genes where changes in ploidy can obscure a phenotype. In our CRISPR-Cas13b mediated knockdown studies, while at much lower levels, mRNA is still produced (Fig. 2, Fig. 3, Fig. 4B) but yet this decrease in expression can be associated with clear phenotypes (Fig. 2, Fig. 3, Fig. 4A). This would be reminiscent of heterozygous gene knockouts that are also often associated with phenotypes (Mukherjee et al., 2009). Since resistance levels reported are low (Fig. 3, Fig. 4), an improved Cas13b system could produce a more robust phenotype but yet incomplete knockdowns hence more easily highlighting genes involved in resistance including essential genes.

Two areas that could benefit from further improvements for the Cas13b system are related to the Cas13 endonuclease itself and the production of optimal gRNAs. We are currently using an episomal version of Cas13b (Fig. 1A) and it would be interesting to test if increased expression of Cas13b (e.g. by integrating the gene into the rRNA locus) would lead to more effective knockdowns. Although Cas13b from *Prevotella* spp. appears to be one of the most effective nucleases in mammalian cells (Cox et al., 2017; Smargon et al., 2017), other orthologs have been characterized more recently and may be effective in *Leishmania*, such as Cas13d (CasRx) (Mahas et al., 2019), or Cas13bt3 (also known as Cas13X.1), one of the smallest Cas13 with 775 residues and already used for RNA knockdowns in mammalian cells (Xu et al., 2021; Kannan et al., 2022; Nakagawa et al., 2022), or finally, a type-III CRISPR–Cas, Cas7–11, targeting RNAs cutting at two unique locations with crRNA sequences between 22 and 31 nucleotides (Özcan et al., 2021; Van Beljouw et al., 2021). A dead (d)Cas13 protein has been described (Huang et al., 2023; Xu et al., 2023) and could be used to modulate the translation of targeted mRNAs. Proteins have already been identified that can modulate the activity of Cas13b and their use could influence the knockdown efficiency. For example, the regulatory accessory proteins Csx27 and CsX28 modulate, respectively, negatively and positively the RNAse activity of Cas13b (Smargon et al., 2017). Similarly, Csx29 has an inhibitory effect on RNAse activity of Cas7-11 (Özcan et al., 2021). Another approach for modulating the activity of Cas13 would be to optimize an inducible system such as the use of a light-modulated split Cas13b/dCas13b system (Xu et al., 2023), an Inducible SpliT Cas13 Orthologs and Exogenous Ligands (CRISTAL) (Ding et al., 2024) or using strategies for conditional destabilization of a Cas13b fusion protein, a strategy that has be shown to work in *Leishmania* (Madeira Da Silva et al., 2009).

The selection of gRNAs against target genes and their proper expression are equally important for an optimal CRISPR-Cas13b system. For the *LUC* gene, we tested 2 crRNAs where one worked better, and out of 9 crRNAs targeting *MT*, two did not lead to the knockdown of the targeted gene. One important parameter for knockdown efficiency is the length of the crRNA. Several studies have tested crRNAs ranging from 15 to 84 nucleotides to determine the optimal crRNA length for each member of the Cas13 family with an initial consensus of crRNAs around 30 nucleotides (Cox et al., 2017; Smargon et al., 2017; Huynh et al., 2020; Xu et al., 2021, 2023). In this study, the crRNA length for the three targeted genes was respectively of 26 and 28 nucleotides (Table S1). However, a recent study has suggested that shorter crRNAs (22–25 nucleotides) for Cas13b are even better (Liu et al., 2023). Interestingly, the *LUC* knockdowns had a phenotype without further selection with hygromycin B/puromycin while the crRNAs against *MT* or *AQP1* were phenotypic only after the additional selection with MF or SbIII, possibly to select the few cells within a population that had optimal Cas13b and gRNA expression. Since the crRNAs for *LUC* were 2 nucleotides smaller than the ones for *MT,* it will be worth testing the activity of shorter crRNAs in further Cas13 development in *Leishmania,* although a 26 nt gRNA was used for *AQP1* and we still had to use drug selection first. In addition to the length of the crRNAs, its accessibility to the target RNA sequence was shown to matter (Abudayyeh et al., 2016; Smargon et al., 2017; Bandaru et al., 2020; Wessels et al., 2020). The use of advanced bioinformatics tools based on RNA structure prediction, such as the siRNA design tool RNAxs (Tafer et al., 2008) or the EvryRNA platform http://EvryRNA.ibisc.univ-evry.fr/(Engelen and Tahi, 2010), could be helpful in selecting crRNAs for regions not predicted to have complex secondary structures. With the caveat that RNA structure is only a prediction, it is noteworthy that the two *MT* guides, *MT*-2 and *MT*-5, that have failed to lead to MF resistant parasites are the crRNA sequences that, according to predictions, would recognize sequences with complex secondary structures, either in the 5’ extremity or by steric hindrance (Fig. S5). The gRNAs are a combination of the crRNA and a sequence recognized by Cas13b. We used a 88 bp sequence with direct repeats (Smargon et al., 2017) but shorter sequences with direct repeats such as a 36 bp sequence (Cox et al., 2017; Slaymaker et al., 2019) have been shown to be effective for knocking down gene expression in mammalian cells, and these shorter versions could be tried in *Leishmania* to assess whether they could improve the Cas13b-mediated knocking down of gene expression.

In summary, we have shown that the CRISPR-Cas13 system is effective for gene knockdowns in the parasite *Leishmania*. This has the further advantages that it does not edit the DNA, so effects could be transient, if desired, and represents a valuable tool to assess phenotypes of essential genes. Further developments would potentially allow this strategy to be highly applicable to various aspects of *Leishmania* biology including large genomic screens.

## 5 Conclusion

This study represents the first application of a CRISPR-Cas13 system for gene knockdowns in *Leishmania* parasites. This new tool holds potential to enable specific knockdowns of coding and non-coding RNAs, facilitate transitory gene inactivation, and can be used for high-throughput applications. This new molecular research tool for silencing gene expression in protozoan parasites could pave the way for new advances in combating parasitic diseases.

## CRediT authorship contribution statement

**Marine Queffeulou:** Writing – review & editing, Writing – original draft, Investigation, Formal analysis, Data curation, Conceptualization. **Raouia Fakhfakh:** Writing – review & editing, Investigation, Formal analysis, Conceptualization. **Fereshteh Fani:** Writing – review & editing, Investigation, Formal analysis, Conceptualization. **Alex Dos Santos:** Writing – review & editing, Investigation. **Gabriel Reis Ferreira:** Writing – review & editing, Investigation. **Sophia Bigot:** Writing – review & editing, Investigation. **Chantal Godin:** Writing – review & editing, Investigation. **Philippe Leprohon:** Writing – review & editing, Supervision, Software, Investigation, Formal analysis, Conceptualization. **Barbara Papadopoulou:** Writing – review & editing, Supervision, Funding acquisition, Conceptualization. **Marc Ouellette:** Writing – review & editing, Writing – original draft, Supervision, Methodology, Investigation, Funding acquisition, Formal analysis, Conceptualization.

## Declaration of competing interest

The authors declare that they have no known competing financial interests or personal relationships that could have appeared to influence the work reported in this paper.
