# Supplementary material for: CRISPR-Cas13b mediated gene knockdowns in Leishmania infantum
Source: Int J Parasitol Drugs Drug Resist. 2025 Dec 5;29:100629. doi: 10.1016/j.ijpddr.2025.100629 (PMC12743515; doi:10.1016/j.ijpddr.2025.100629)
Supplement: Multimedia component 1 [file mmc1.docx]

Supplementary figures and tables to **"**CRISPR-Cas13b mediated gene knockdowns in *Leishmania infantum* **"**

Marine Queffeulou, Raouia Fakhfakh, Fereshteh Fani, Alex Dos Santos, Gabriel Reis Ferreira, Sophia Bigot, Chantal Godin, Philippe Leprohon, Barbara Papadopoulou and Marc Ouellette


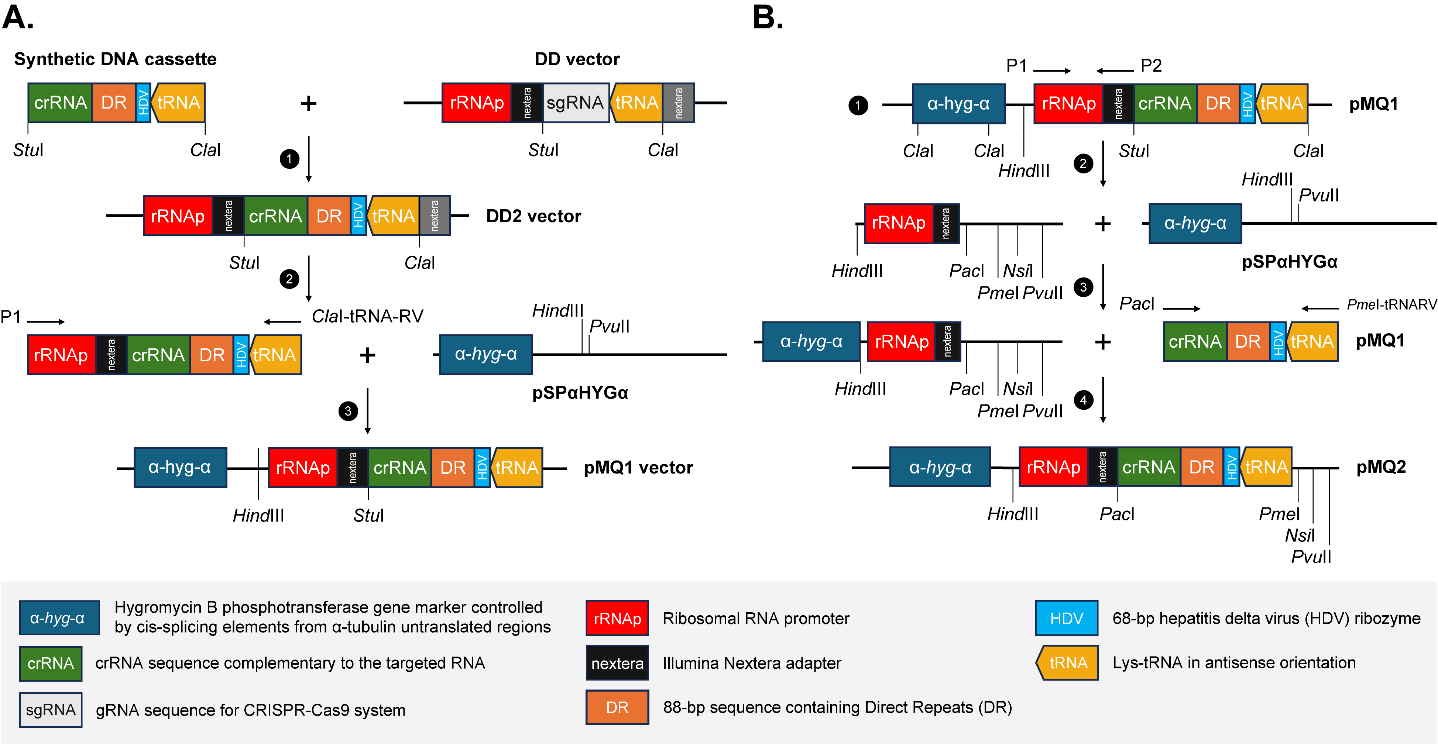


**Figure S1. Construction of gRNA-expressing vectors pMQ1 and pMQ2. A. Generation of pMQ1.** A 361-nt synthetic DNA cassette was synthesized and contains a crRNA sequence (variable sequence), linked to an 88-nt direct repeat (DR) sequence (orange box) derived from *Bergeyella zoohelcum* ATCC 43767 (49), with a hepatitis delta virus (HDV) ribozyme sequence (light blue box) and a tRNA in antisense orientation (orange box) acting as transcription terminators. This DNA cassette was first integrated into a *Stu*I and *Cla*I site of the DD vector (23) under the control of a ribosomal RNA promoter (rRNAp; red box) and positioned between two Nextera adapters (black and dark grey boxes). Two specific gRNAs against the *LUC* gene were amplified by PCR with primers A or B and *Cla*I-tRNA-RV (Table S1) and inserted into a *Stu*I-*Cla*I digested vector, leading to DD2. The complete gRNA expression cassette was amplified by PCR and integrated into the *Pvu*II site of the high copy number vector pSPα-HYG-α (51) to generate pMQ1. **B. Generation of pMQ2.** The ribosomal RNA promoter and its Nextera adapter DNA of pMQ1 was amplified by PCR (P1 and P2 primers; Table S1), incorporating multiple unique restriction sites including *Pac*I and *Pme*I. This PCR product was cloned into the *Hin*dIII and *Pvu*II pSPα-HYG-α vector. Each gRNA against the *MT* gene was then amplified by PCR, using a forward primer consisting of *Pac*I followed by a 28-nt unique crRNA sequence and 20-nt partial DR (1 to 9; Table S1) coupled with a unique reverse primer corresponding to 21 nt of the tRNA terminator (*Pme*I-tRNARV). The PCR fragment was cloned into the *Pac*I and *Pme*I sites of pSPα-HYG-α-rRNAp vector to lead to pMQ2.





**Figure S2.** **Growth kinetics of *Leishmania infantum transfected with a* Cas13b-expressing vector.** Growth curve of *L. infantum* WT (☐); Cas13-expressing *L. infantum* (●) (in absence of puromycin), and culture medium (SDM) (▲). Parasite Growth curves were realized using an automated incubation system (Biospa, BioTek) integrated to a Cytation 5 multimode reader (BioTek). Parasites (2 × 10^6^ cells) were cultured in 6-well Falcon plates conatining 3 ml of SDM 79 medium supplemented with 10% heat-inactivated fetal bovine serum, 10 µM biopterin, and 5 µg/ml of hemin. Growth was monitored by measuring optical density at 600 nm every 4 hours over a period of 188 hours using Cytation5 reader. Error bars represent standard deviation from replicates.


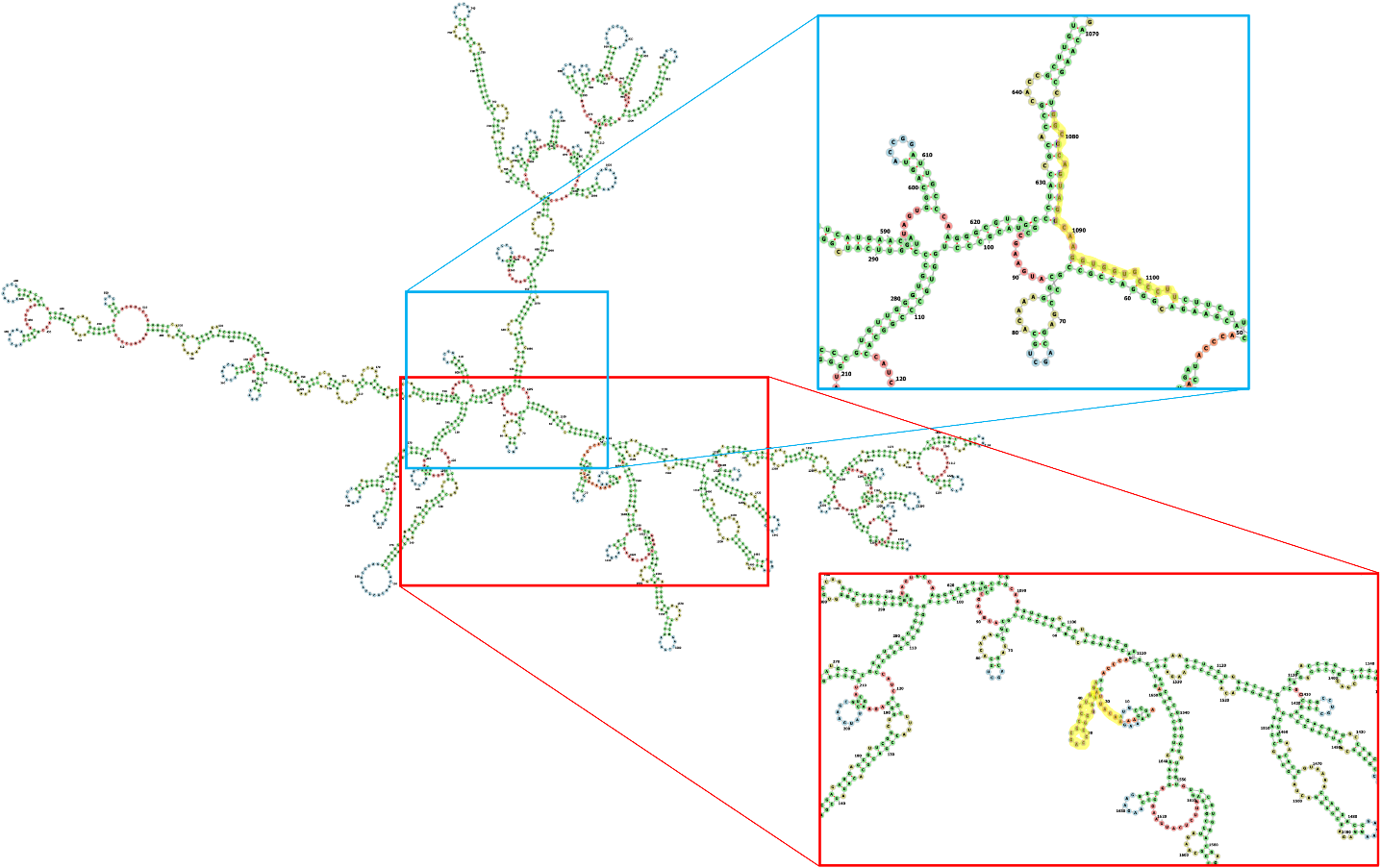


**Figure S3.** **Secondary structure prediction of the firefly luciferase mRNA and its interactions with two crRNA sequences**. The firefly luciferase secondary structure prediction was generated by ViennaRNA Web Services at a temperature of 26°C. The crRNA sequences are highlighted in yellow and were designed either at the beginning of the gene (red square; LUC-A) or in its middle (blue square; LUC-B). Reverse complement primers for gRNA amplification are listed in Table S1.





**Figure S4.** **Replication of Cas13b-mediated gene knockdown of the firefly luciferase gene using different gRNA sequences.** Two crRNA sequences were designed to target the luciferase gene: LUC-A corresponds to a sequence at the beginning of the gene (from position 15 to 41) whereas LUC-B is complementary to a region in the middle of the gene (nucleotides 1077 to 1103). Luciferase assay were done on *L. infantum* strains with a luciferase gene (*LUC*) integrated into the genome (*LINF_360047000;* aminomethyltransferase - mitochondrial)*,* harboring the gRNA-LUC-A expression-vector and transfected without (1) or with (2) the Cas13b-expression vector, or harboring the gRNA-LUC-B expression-vector with a Cas13b-expression vector (3). Luminescence analysis was done with 1 x 10^7^ cells of *L. infantum* harboring a luciferase gene using a 96-well plate luminometer system. Data represent the mean of relative light units (RLUs) recorded from 2 independent experiments in technical triplicates. Error bars represent standard deviation. Significance was denoted as **p≤0.001 and ***p≤0.0001.


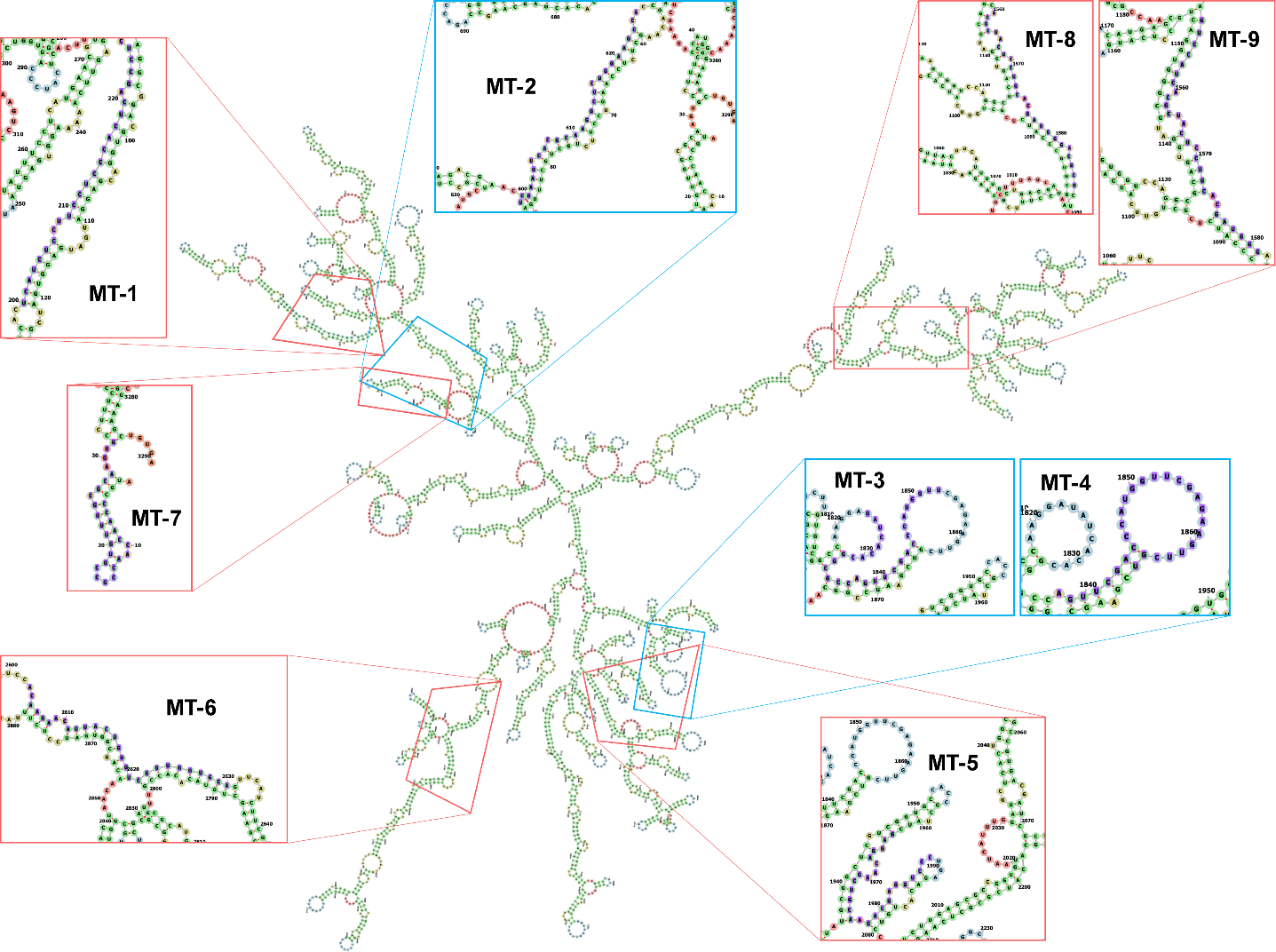


**Figure S5.** **Secondary structure prediction of the Miltefosine transporter (*LINF_130020800*; *MT*) mRNA and its interactions with nine different crRNAs.** *MT* mRNA secondary structure prediction was generated by ViennaRNA Web Services at a temperature of 26°C. The crRNA sequences were designed along the *MT* mRNA avoiding as much as possible putative secondary structures as stemloops or pseudoknots. Some crRNAs were similar; MT-3 and MT-4 shared 16 nucleotides; MT-8 and MT-9 shared 21 nucleotides. MT-2 and MT-5 sequences appeared to be less accessible for Cas13b protein (either in 5’ extremity or by steric hindrance). Reverse complement primers for the amplification of the gRNA are listed in Table S1.





**Figure S6. CRISPR-Cas13b mediated *MT* gene knockdown and resistance phenotype to miltefosine.** *L. infantum* recombinant cells expressing the Cas13b vector or not were transfected with pMQ plasmids encoding the gRNAs MT1-MT-9 or LUC-A. Cells expressing both Cas13b and *MT*’s gRNA could be selected with 5X EC_50_ miltefosine, except MT-2 and MT-5. The EC_50_ values were calculated by monitoring their growth when incubated in presence of gradual MF concentrations at 600 nm at the 72h end-point or when the baseline (cells without MF) reached an O.D of 0.5. Cells not expressing Cas13b could not be selected with 5X EC_50_ miltefosine and their EC50s were measured after transfections of the MQ MT or LUC-A plasmids. The presence or not of Cas13b and the presence of distinct *MT* gRNAs are indicated below the graph. The mean and standard deviation of a minimum of two independent experiments in technical triplicates are shown. Significance was denoted as **p≤0.001 and ***p≤0.0001.





**Figure S7. Independent CRISPR-Cas13b mediated knockdown of the *L. infantum* *MT* gene using a MT-1 gRNA.** *L. infantum* recombinant cells expressing the Cas13b vector and transfected with pMQ plasmids encoding the gRNA against MT1 (primer 1 in Table S1) grown without miltefosine selection (⭘, a) or with 40 uM of miltefosine for 3 passages followed by 3 passages without miltefosine (●, b). **A. Miltefosine susceptibility**. The EC_50_ values were calculated by monitoring their growth when incubated in presence of gradual miltefosine concentrations, at 600 nm at the 72h end-point. The mean and standard deviation of at least three biological replicates are shown. **B. Monitoring *MT1* mRNA expression**. Quantitative real-time RT-PCR was conducted to measure *MT* gene expression, normalized against constitutively expressed *GAPDH* gene. RNA extractions were performed from mid-log phase promastigotes in biological triplicates, with RNA expression measurements assessed in at least three independent experiments with technical triplicates and represented here by the mean. Standard deviation is represented by error bars and significance was denoted as ***p≤0.0001.

**Table S1 : List of primers and crRNA sequences used for the CRISPR-Cas13 experiments**

| **Type** | **Name or id** | **Sequence** |
| --- | --- | --- |
| **Primers pSPrRNAp-gRNA** | P1. HindIII-rRNApFW | GGC**AAGCTT**TTGCTGTGTGCGTGTGTATGTGG |
|  | P2. PvuII-NsiI-PmeI-PacI-nextera1-RV | GGC**CAGCTGATGCATGTTTAAACTTAATTAA**CTGTCTCTTATACACATCTGACGCTGC |
|  | ClaI- tRNARV | TAATCGATAAGCATTCCTAGCTCAGTC |
|  | PmeI-tRNARV | GCCGTTTAAACGCATTCCTAGCTCAGTCGGTAG |
| **Primers for amplifying the gRNAs** | A. crRNAluc1revStuIFW | cg**AGGCCT**gcaatggcgctgggcccttcttaatgttGTTGGAACTGCTCTCATTTTAT |
|  | B. crRNAluc2revStuIFW | cg**AGGCCT**gcaagggcaccaccttgcctactgcgccGTTGGAACTGCTCTCATTTTAT |
|  | 1.crRNALiMTrevPacIFW | GG**TTAATTAA**GAGGCTGAGTGGGAGGAAGGAGATGAGTGTTGGAACTGCTCTCATTTTAT |
|  | 2.crRNALiMTrevPacIFW | GG**TTAATTAA**TGGCTTCCAGAGCCTTGCGTGACTTCAGGTTGGAACTGCTCTCATTTTAT |
|  | 3.crRNALiMTrevPacIFW | GG**TTAATTAA**CCATGGGTCGAACTGGCGCCGTGTGATAGTTGGAACTGCTCTCATTTTAT |
|  | 4.crRNALiMTrevPacIFW | GG**TTAATTAA**ACGAACTTCTCGAACCATGGGTCGAACTGTTGGAACTGCTCTCATTTTAT |
|  | 5.crRNALiMTrevPacIFW | GG**TTAATTAA**CAGGGACCTCGTCTTGCAGCTTGTCCTCGTTGGAACTGCTCTCATTTTAT |
|  | 6. crRNALiMTrevPacIFW | GG**TTAATTAA**TGCACCACCGACACAGTAATGTTCTTGTGTTGGAACTGCTCTCATTTTAT |
|  | 7. crRNALiMTrevPacIFW | GG**TTAATTAA**CACTTGCGCCAACACGGCGGTTGGTTGGGTTGGAACTGCTCTCATTTTAT |
|  | 8.crRNALiMTrevPacIFW | GG**TTAATTAA**TGCGCGTCCCATCGTGCAGGAGTAGCGTGTTGGAACTGCTCTCATTTTAT |
|  | 9.crRNALiMTrevPacIFW | GG**TTAATTAA**CCCATCGTGCAGGAGTAGCGTGATGGACGTTGGAACTGCTCTCATTTTAT |
|  | 10.crRNALiAQP1revPacIFW | GG**TTAATTAA**GGAAGCAAGGCCTTTTGGGCGTCGTCGTTGGAACTGCTCTCATTTTAT |
| **Primers used for Cas13b-expressing vector** | C. XbaICas13bFW | GCC**TCTAGA**ATGAACATCCCCGCTCTGGT |
|  | D. HindIII cas13b-HIVNES_HAtagRV | GCC**AAGCTT**TTAGGCATAGTCGGGGACAT |
| **Primers for RT-PCR** | qPCR MT Li263 FW | TCTCGTCTACTCCGCCATTC |
|  | qPCR MT Li263 RV | CCAACGTAGAGGAGCAGGAA |
|  | qPCR GAPDH Li263 FW | GCAGACGTACATGAAGGGCA |
|  | qPCR GAPDH Li263 FW | CGGGTAGGTTGTTCTGCAGT |
|  | qPCR AQP1 Li263 FW | CGAGTATGTTGCCGAGTTCTT |
|  | qPCR AQP1 Li263 FW | CATGTAGCTGGAGTTGGACTG |
| **Synthetic cassette as template for initial gRNA expression-cassette in DD vector** | **StuI** - **crRNA sequence (variable)**-**DR**-**HDV**-tRNA - **ClaI** | CGG**AGGCCT**GC**GCAACACGGTTCAGCCCTTCAAGGACGACA**GTTGGAACTGCTCTCATTTTATTCGTGAAGTTTTTATTTGTTTTCAAAGGAACTCATGAATACAGAGTATTTGGAGGGTAATAACAAC**GGCCGGCATGGTCCCAGCCTCCTCGCTGGCGCCGGCTGGGCAACATGCTTCGGCATGGCGAATGGGAC**CCCACAAAAACGGCGGCGTGCGGCCCCGCCGAAGCCCCCCCCCTTCTTCTCTACTAAAGGGGGTCCGGCCGGGGAAAAAACGCACTCCGTGGGGATCGAACCCACGACCACACGGTTAAGAGCCGTGCGCTCTACCGACTGAGCTAGGAATGCTT**ATCGAT**GGC |
| **Primers for Northern-blot probes** | 5'.**NcoI**-luc | TA**CCATGG**ACATGGAAGACGCCAAAAACA |
|  | 3'.**EcoNI**-luc | AT**CCTAATTAAG**GCAATTTGGACTTTCCGCCC |
